# A Multifunctional Cationic Waterborne Polyurethane System with High Fire-Safety and Antibacterial Performance Enabled by Phosphorous Acid-Protonated Chitosan

**DOI:** 10.3390/biomimetics11060384

**Published:** 2026-06-01

**Authors:** Xin-Yu Tian, Zhen-Guo Zhao, Peng Chen, Yan-Peng Ni

**Affiliations:** Institute of Functional Textiles and Advanced Materials, College of Textiles & Clothing, Qingdao Key Laboratory of Flame-Retardant Textile Materials, Shandong Key Laboratory of Polymeric Materials Recycling and Upcycling, National Engineering Research Center for Advanced Fire-Safety Materials D & A (Shandong), Qingdao 266071, China

**Keywords:** waterborne polyurethane, chitosan derivative, flame retardancy, antibacterial

## Abstract

Waterborne polyurethane (WPU) is widely used in flexible films and textile finishing, but its intrinsic flammability, severe melt dripping, and sensitivity to polar additives restrict its fire-safe applications. Herein, a phosphorous acid-protonated chitosan (PCS) was designed as an emulsion-adaptable bio-based modifier and incorporated into cationic WPU via a facile aqueous blending route, yielding transparent multifunctional composite films and flame-retardant textile coatings. Unlike conventional flame-retardant WPU systems that rely on reactive monomers or suffer from poor emulsion compatibility, this work proposes an emulsion-compatible strategy based on PCS, enabling the simultaneous integration of dispersion stability, flame retardancy, and antibacterial functionality within a single system. PCS could be stably accommodated in the WPU latex without visible precipitation or demulsification after centrifugation, and the resulting films preserved a continuous matrix structure with uniformly distributed PCS-rich nanodomains. Rheological analyses revealed that the polar groups of PCS established strong intermolecular associations with urethane segments, strengthening the physical network. The char residue at 700 °C increased from 0.7 wt% for neat WPU to 32.7 wt% for WPU/PCS-5. Meanwhile, WPU/PCS-5 achieved a limiting oxygen index of 35.4% and a UL-94 V-0 rating, while its peak heat release rate and total heat release were reduced by 73.4% and 41.8%, respectively. The composite films also showed nearly complete antibacterial efficiency against *Escherichia coli* and *Staphylococcus aureus*. As a textile coating, WPU/PCS-5 enabled immediate self-extinguishing of cotton fabric, increased the limiting oxygen index from 18.5% to 27.2%, and reduced the damaged length from 30.0 to 11.0 cm. This work demonstrates that an emulsion-compatible strategy based on PCS can effectively integrate dispersion stability, fire safety, multifunctionality, and coating applicability into WPU materials.

## 1. Introduction

Waterborne polyurethane (WPU) has attracted considerable attention as an environmentally friendly polymer material because it uses water as the primary dispersion medium [1,2,3], reducing the reliance on volatile organic solvents during coating and finishing processes. Owing to its excellent flexibility, adhesion, abrasion resistance, film-forming ability, and structural tunability, WPU has been widely applied in coatings, adhesives, leather finishing, flexible films, and textile finishing applications [4,5]. Nevertheless, the flame retardancy of WPU remains a critical issue. Its organic polymer backbone is prone to thermal decomposition and combustion, and low-viscosity liquid fragments generated during thermal degradation may cause severe dripping, which can accelerate flame spread and ignite surrounding combustible substrates. Therefore, improving the flame retardancy and anti-dripping behavior of WPU is essential for broadening its application in fire-safe coatings and flexible textile-related materials.

Unlike conventional polymer solutions, WPU is a typical colloidal latex system. Its dispersion stability is closely governed by the interfacial state of latex particles, including particle size, surface charge, electrostatic repulsion, hydrophilic segment distribution, and viscosity. When external functional additives, especially those containing abundant polar groups or ionic sites, are introduced into WPU, the original balance among latex particles may be disturbed, potentially leading to changes in particle size distribution, increased viscosity, particle aggregation, sedimentation, or even demulsification [6,7,8]. Consequently, the construction of flame-retardant WPU composites cannot be evaluated solely by the intrinsic flame-retardant efficiency of the additive. The compatibility with the charged emulsion system, as well as the ability to maintain stable dispersion and continuous film formation, should also be considered as essential criteria. This colloidal sensitivity of WPU further complicates the development of flame-retardant formulations, as many conventional flame retardants that perform well in solvent-based or bulk polymer systems cannot be directly transferred to aqueous polyurethane emulsions.

In recent years, various element-containing flame retardants have been developed to improve the fire safety of WPU [9,10,11]. For example, Feng et al. [12] prepared a phosphorus-, nitrogen-, and silicon-containing flame-retardant WPU through a one-step synthetic approach, achieving improved flame retardancy. Tong et al. [13] reported a non-ionic flame-retardant WPU and noted that although reactive flame retardants have been widely explored, insufficient char formation and melt dripping during combustion remain persistent challenges. These studies confirm the significance of molecular design in flame-retardant WPU, but many reported approaches still rely on reactive synthesis, specially designed monomers, or non-renewable flame-retardant components [14]. More critically, these existing strategies often fail to adequately address the two intrinsic shortcomings of WPU, namely poor char-forming ability and severe melt dripping during combustion. In this context, the direct incorporation of bio-based functional additives offers a simpler and more sustainable route, provided that the stability of the waterborne emulsion can be preserved [15].

Bio-based flame retardants have garnered increasing attention because of their renewable origin, structural diversity, and potential environmental benefits [16,17,18]. Among natural polymers, chitosan is particularly attractive as a bio-based platform. Because it contains abundant amino and hydroxyl groups, possesses intrinsic film-forming ability, and can provide antibacterial functionality [19]. Moreover, phosphorus-containing groups can be introduced onto chitosan chains through chemical modification, improving flame-retardant performance. For example, Zhao et al. [20] prepared a phosphorus-containing chitosan derivative via one-step protonation and applied it to polyester fabrics, achieving excellent flame retardancy, anti-dripping behavior, and antibacterial performance. From a materials-design perspective, chitosan-derived phosphorus-containing additives are appealing because they combine phosphorus-based char-promoting capability with the inherent film-forming and antibacterial functions of chitosan [21,22]. The biomimetic design of this work is inspired by the phosphorus-nitrogen synergistic chemical defense strategies widely found in nature. Biomolecules such as DNA (containing a phosphate backbone and nitrogenous bases) and phytic acid (the primary phosphorus storage form in plants) exhibit dual functionalities: upon heating, they form protective phosphorus-containing char layers via phosphorus-catalyzed charring, providing flame-retardant effects; meanwhile, they or their derivatives also demonstrate antibacterial activity (e.g., DNA fragments disrupt bacterial cell membranes through electrostatic interactions, and phytic acid chelates metal ions to inhibit bacterial growth). This natural phosphorus-nitrogen synergistic mechanism inspired us to design an artificial system combining a bio-based nitrogen source (chitosan) with a phosphorus source (phosphorous acid), yielding phosphorous acid-protonated chitosan (PCS) via a simple protonation reaction. We have previously demonstrated that such PCS can simultaneously impart flame retardancy and antibacterial properties to PVA composites through hydrogen-bonding and interfacial interlocking [23]. Moreover, the phosphorus-nitrogen synergistic strategy has also been successfully applied to PLA [24] and PET [25] systems, confirming its broad effectiveness in enhancing fire safety. In the present work, PCS is introduced into a cationic WPU emulsion, where it forms uniformly dispersed nanodomains within the WPU matrix, endowing the material with excellent flame retardancy and antibacterial performance simultaneously.

The target applications of such multifunctional coatings include interior decorative textiles, furniture upholstery, and dry-use protective clothing—scenarios where both fire safety and antibacterial protection are essential. In these environments (e.g., public seating, office furnishings, and industrial workwear), materials are expected to resist ignition and flame spread while also minimizing bacterial contamination. The integration of both properties into a single coating system using a bio-based additive (PCS) offers a streamlined and sustainable solution. Despite these advantages, the adaptation of such chitosan-derived additives to charged WPU emulsions has rarely been examined. Given the high sensitivity of WPU to changes in latex–particle interactions, whether PCS can be effectively incorporated without compromising emulsion stability or film quality remains an open question [26,27].

In this work, PCS was introduced into cationic WPU through a facile aqueous blending strategy to construct multifunctional WPU/PCS-x composite films and textile coatings. This study focuses primarily on evaluating the compatibility of PCS with the WPU emulsion and the resulting film performance. The dispersion stability, microstructure, optical transparency, intermolecular interactions, and rheological behavior of the composite systems are investigated to assess the effect of PCS incorporation. Furthermore, the thermal decomposition behavior, flame retardancy, antibacterial activity, and textile-coating applicability of the WPU/PCS system are systematically examined. This study aims to provide a practical, emulsion-compatible strategy for developing bio-based flame-retardant WPU materials, thereby demonstrating that a compatibility-first design principle can achieve synergy among dispersion stability, fire safety, and multifunctionality.

## 2. Experiment Section

### 2.1. Materials

Isophorone diisocyanate (IPDI), polypropylene glycol 2000 (PPG, Mw = 2000), N-methyldiethanolamine (MDEA), 1,4-Butanediol (BDO), dibutyltin dilaurate (DBTDL) and Chitosan (CS, Mw: 200,000 g·mol^−1^, degree of deacetylation = 85%) were supplied by Macklin Biochemical Co., Ltd. (Shanghai, China).

### 2.2. Synthesis of Cationic WPU

Based on the synthetic protocol reported in our previous work [28], polypropylene glycol 2000 (PPG) was vacuum-dehydrated at 110 °C for 2 h and then cooled to 80 °C. The dehydrated PPG (0.02 mol) was transferred into a four-necked flask, followed by the addition of isophorone diisocyanate (IPDI, 0.12 mol). The reaction was carried out under a nitrogen atmosphere with a condenser. After 10 min of reaction, five drops of dibutyltin dilaurate (DBTDL) were added as the catalyst, and the reaction was continued for another 50 min. Subsequently, N-methyldiethanolamine (MDEA, 0.04 mol) was introduced into the flask to initiate the chain-extension reaction. The viscosity of the reaction mixture gradually increased, and the reaction was maintained for 1 h. Afterward, 1,4-butanediol (BDO, 0.04 mol) was added for further chain extension. During this process, an appropriate amount of acetone was slowly added dropwise to reduce the viscosity of the reaction system. After completion of the chain-extension reaction, the temperature was decreased to 50 °C, and a stoichiometric amount of acid was added to neutralize the tertiary amine groups and render the polyurethane water-dispersible. The reaction was continued for 1 h. The stirring speed was then increased to 1300 r/min, and 300 mL of deionized water was added dropwise into the flask under high-speed stirring. Emulsification was continued for 1 h. Finally, acetone was removed using a rotary evaporator to obtain the waterborne polyurethane dispersion.

### 2.3. Preparation of H_3_PO_3_-Protonated Chitosan Derivative (PCS)

Following a facile, atom-economic and mild strategy reported in the literature [29], a phosphorous acid-protonated chitosan derivative (PCS) was prepared through an acid-base neutralization process between H_3_PO_3_ and the primary amino groups on chitosan chains. In brief, H_3_PO_3_ (3 g) was first dissolved in deionized water, after which chitosan powder was gradually added according to a molar ratio of 1:1 between H_3_PO_3_ and the amino groups in the chitosan repeating units. The suspension was continuously stirred at ambient temperature until a homogeneous solution was obtained, affording PCS solutions with the desired concentration.

### 2.4. Preparation of WPU/PCS-x Composite Films

A series of WPU/PCS-x composite films were fabricated through an aqueous blending and casting method. Briefly, the PCS solution was added dropwise into the WPU dispersion under continuous stirring to obtain uniform WPU/PCS mixed dispersions with PCS contents of 30 wt.%, 40 wt.%, and 50 wt.% relative to the total solid content. The resulting composite films were designated as WPU/PCS-3, WPU/PCS-4, and WPU/PCS-5, respectively. The mixtures were further stirred at room temperature to promote sufficient dispersion and interfacial interaction between PCS and WPU. After degassing, the resulting dispersions were poured into polytetrafluoroethylene molds and dried at ambient temperature to form continuous films. The films were subsequently dried in an oven to remove residual moisture.The formation mechanism of the WPU/PCS composite system is illustrated in Figure S1.

### 2.5. Characterization

The colloidal stability of the WPU/PCS emulsions was assessed using an accelerated centrifugation test. The emulsions were centrifuged at 3000 rpm for 20 min, and samples without visible phase separation, sedimentation, or demulsification after centrifugation were considered to possess storage stability for more than 6 months.

The particle size distribution and zeta potential were determined using a Malvern Zetasizer Nano ZSE analyzer (Malvern Panalytical Ltd., Worcestershire, UK) after dilution with deionized water.

The optical transmittance of the films in the visible region was recorded on a Shimadzu UV-2700 UV-Vis spectrophotometer (Shimadzu, Kyoto, Japan).

The morphology of the WPU/PCS latex particles was examined by transmission electron microscopy (TEM) using a JEM-F200 microscope (JEOL, Tokyo, Japan). Prior to observation, the WPU/PCS dispersion was diluted with deionized water to a solid content of 1 wt%, and a small droplet was deposited onto a copper grid and dried at room temperature.

FT-IR spectra were acquired on a Nicolet iS 50 spectrometer (Thermo Fisher Scientific, Madison, WI, USA) over the range of 400–4000 cm^−1^ at a resolution of 4 cm^−1^ with 32 scans, using the attenuated total reflectance (ATR) mode.

Dynamic oscillatory frequency sweep tests were conducted on an Anton Paar MCR302 rheometer (Anton Paar GmbH, Graz, Austria) equipped with a parallel-plate geometry to evaluate their frequency-dependent viscoelastic properties.

Thermogravimetric analysis (TGA) was performed on a TGA5500 instrument (TA Instruments, New Castle, DE, USA) under a nitrogen atmosphere at a heating rate of 10 °C/min.

Microscale combustion calorimetry (MCC) was carried out on an FAA microcalorimeter under a nitrogen/oxygen atmosphere. Samples were heated from 50 to 750 °C at 2 °C·s^−1^.

The UL-94 vertical burning test was conducted on a TTech-GBT2408 horizontal and vertical combustion tester (TESTECH, Suzhou, China) in accordance with ASTM D3801 standard [30].

The limiting oxygen index (LOI) was measured with a TTech-GBT2406-2 tester (TESTECH, Suzhou, China) following ASTM D2863-97.

Cone calorimetric tests (CCT) were carried out on a calorimeter (Fire Testing Technology, West Sussex, UK).

The antibacterial activity of the WPU-PCS composite films was evaluated using Escherichia coli (*E. coli*) at 10^8^ CFU/mL and Staphylococcus aureus (*S. aureus*) at 10^7^ CFU/mL. Film samples were incubated with bacterial suspensions under oscillation for 18 h at 37 °C. After treatment, suspensions were serially diluted 101–104, and 0.25 mL of each dilution was plated onto agar and incubated for 24 h. Colonies on plates with 30–300 CFU were counted, and the antibacterial rate (%) was calculated as

$$Y=\frac{W_{t}-Q_{t}}{W_{t}}\times 100\%$$

where $W_{t}$ is the average number of viable colonies in the blank sample after 18 h of shaking incubation, $Q_{t}$ is the average number of viable colonies in the antibacterial sample, and Y is the antibacterial rate of the sample (%).

## 3. Results and Discussion

### 3.1. Emulsion Stability, Compatibility, and Transparency

**Figure 1:**
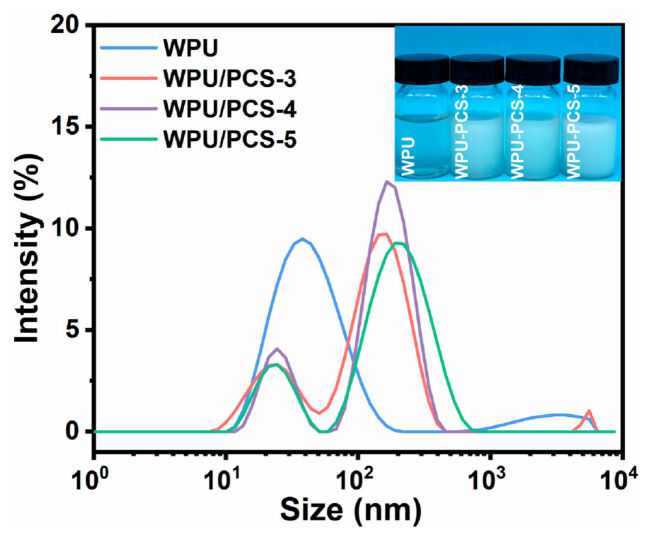
Particle Size Distribution Plot of WPU/PCS-x composite emulsion.

To evaluate the colloidal stability and particle-size distribution of the WPU/PCS-x composite emulsions, dynamic light scattering and zeta potential measurements were performed, with centrifugation tests used as supplementary evidence [31]. The results are presented in Figure 1 and Table 1. After the incorporation of PCS, the particle-size distribution changed markedly from the narrow single peak characteristic of neat WPU to a distinct bimodal distribution. The smaller-size peak can be assigned to WPU latex particles that were not significantly involved in association, while the larger-size peak is likely related to composite particles formed through the participation of PCS. This bimodal profile indicates that PCS induced particle reorganization within the dispersion. Concurrently, the average particle size increased from 39.4 nm (neat WPU) to 95–100 nm, and the PDI broadened from 0.310 to 0.498–0.565, consistent with the emergence of the larger composite particle population.

Although the particle size increased and the distribution became broader, the absolute zeta potential values of the composite emulsions remained above 30 mV, exceeding the commonly accepted empirical threshold for good electrostatic stability in colloidal systems. This result suggests that the introduction of PCS did not cause significant destabilization of the emulsion. Instead, the composite particles were still able to maintain stable dispersion through sufficient surface-charge repulsion. To further verify the emulsion stability, high-speed centrifugation was conducted. After centrifugation at 3000 rpm for 15 min, no visible sedimentation or flocculation was observed for any sample. These results confirm that PCS can be incorporated into WPU without compromising the colloidal stability required for film formation.

**Figure 2:**
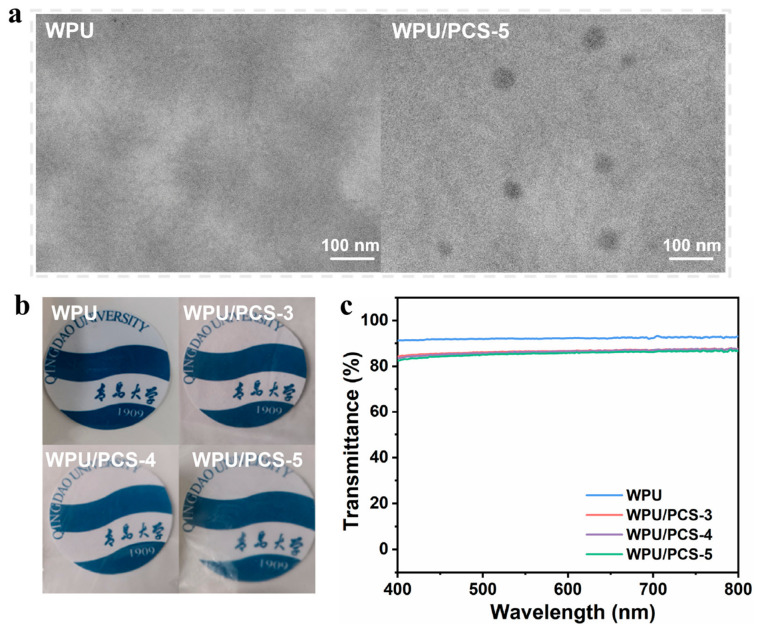
(**a**) TEM of WPU and WPU/PCS-5, (**b**) digital photographs showing the transparency and (**c**) UV-visible transmittance spectra of the composite films.

TEM imaging was employed to examine the microstructure of the composite films. As shown in Figure 2a, neat WPU exhibits a continuous and uniform light-gray matrix with alternating bright and dark microdomains, characteristic of the microphase-separated soft and hard segments. For WPU/PCS-5 composite film, numerous dark nanoscale domains of approximately 30–70 nm were uniformly dispersed throughout the continuous WPU matrix, without macroscopic phase separation or large aggregates. These domains are attributed to the PCS-rich phase. The retention of the inherent WPU microphase-separated morphology, together with the uniform nanoscale dispersion of PCS, indicates that the two components are well compatible rather than simply physically mixed. The good compatibility between PCS and WPU may be primarily associated with the strong intermolecular interactions. More specifically, the abundant hydroxyl, protonated amino, and phosphorus-containing groups of PCS can interact with the urethane linkages and amine-containing segments of WPU through hydrogen bonding and electrostatic interactions. These interactions effectively suppress the self-aggregation of PCS and enable its uniform nanoscale dispersion within the WPU matrix.

As shown in Figure 2b, the films of all the samples exhibited good visual transparency, as the underlying patterns and characters could still be clearly observed through the films. The UV-vis transmittance spectra in Figure 2c further confirmed that all samples maintained high optical transmittance in the visible region of 400–800 nm. The transmittance decreased slightly with increasing PCS content, which is ascribed to enhanced light scattering from the increased population of PCS-rich nanodomains. Nevertheless, the WPU/PCS-x films still remained sufficiently transparent for coating applications, further indicating that the incorporation of PCS did not induce severe aggregation or macroscopic phase separation.

Overall, these findings highlight that PCS can be compatibly integrated into WPU, achieving uniform nanoscale dispersion in the film while retaining the optical transparency essential for coating uses.

### 3.2. Structural Characterization and Hydrogen-Bonding Network Analysis

**Figure 3:**
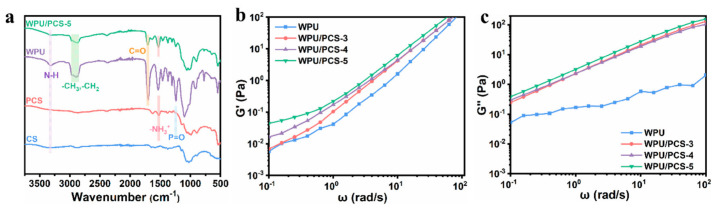
(**a**) FTIR spectrum of PCS, WPU/PCS, frequency dependence of (**b**) G′ and (**c**) G″ of WPU/PCS-x.

As shown in Figure 3a, the FTIR spectrum of PCS displays a characteristic absorption band of protonated amino groups (-NH_3_^+^) in the region of approximately 1526 cm^−1^ and a distinct P=O stretching band near 1250 cm^−1^, which are absent in pristine CS. These spectral features confirm the successful protonation of chitosan by phosphorous acid via acid–base neutralization, indicating the successful synthesis of PCS. The FTIR spectrum of neat WPU exhibits the typical absorption features of polyurethane. The bands at around 3320 and 1700 cm^−1^ are assigned to the stretching vibrations of N-H and C=O groups in urethane linkages, respectively. The absorption peaks in the range of 2860–2970 cm^−1^ correspond to the C-H stretching vibrations of methyl and methylene groups. In the WPU/PCS-x composite film, the characteristic peaks of both WPU and PCS are retained, and the coexistence of hydroxyl, amino, phosphate ester, and urethane functionalities provides abundant sites for intermolecular hydrogen bonding.

To clarify the influence of PCS on the viscoelastic behavior and internal network structure of the WPU dispersion, dynamic oscillatory frequency sweep tests were performed. The steady-shear viscosity of the emulsions as a function of shear rate is shown in Figure S2. As shown in Figure 3b,c, both the storage modulus (G′) and loss modulus (G″) of all the WPU/PCS composite systems exhibited a clear frequency dependence across the tested frequency range, indicating typical viscoelastic behavior [32]. Compared with neat WPU, the incorporation of PCS led to a pronounced increase in both G′ and G″ moduli, and the overall modulus level increased with increasing PCS content. This result suggests that PCS strengthens the interactions among latex particles and enhances the structural association within the dispersion, thereby improving the resistance of the system to deformation. At low frequencies, the composite systems retained a dominant viscous character (G″ > G′), indicating that the internal network can undergo relaxation and flow under prolonged deformation. This behavior is indicative of a dynamic physical crosslinking network sustained by reversible intermolecular interactions rather than permanent covalent crosslinks. The abundant hydroxyl, protonated amino, and phosphite groups on PCS provide numerous hydrogen-bonding sites that can interact with the urethane segments of WPU, thereby enhancing both inter-particle associations and chain–segment interactions within the composite system. In addition, the protonated amino groups of PCS and the ionic centers within WPU may participate in electrostatic interactions, further contributing to the physical crosslinking network. As the PCS content increases, a progressively denser network is established, leading to an enhanced elastic response and improved structural integrity. The increase in G′ in the low-frequency region further indicates that the composite dispersions retain a more stable internal structure over extended relaxation times, which is beneficial for both dispersion stability and uniform film formation.

Overall, the rheological results demonstrate that PCS is effectively integrated into the WPU system through a dynamic physical network involving both hydrogen-bonding and electrostatic interactions. This network enhances the structural stability and viscoelastic response of the WPU/PCS-x composite dispersions, which is critical for uniform film formation and consistent coating quality. These rheological characteristics provide a mechanistic basis for the satisfactory film quality and coating applicability of the WPU/PCS system.

### 3.3. Thermal Decomposition of WPU/PCS-x Composite Films

**Figure 4:**
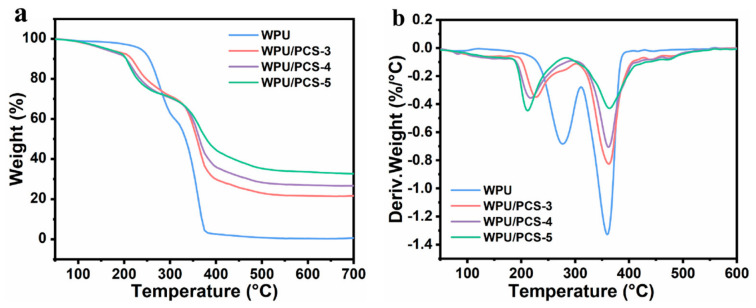
(**a**) TGA curve and (**b**) DTG curve of WPU/PCS-x composite films.

The thermal decomposition behavior of the WPU/PCS composite films was evaluated by TGA. The corresponding TGA and DTG curves are shown in Figure 4, and the detailed thermal parameters are summarized in Table 2. All samples exhibited two principal degradation steps. For neat WPU, the first degradation stage was mainly associated with the cleavage of urethane linkages in the hard segments and the decomposition of partial side groups, whereas the second stage was attributed to the further degradation of soft segments and the continuous decomposition of intermediate carbonaceous structures. After the incorporation of PCS, the thermal degradation behavior of the composite films changed noticeably. Both the initial decomposition temperature (*T*_5%_) and the first maximum decomposition rate (*T_d_*_1*max*_) shifted to lower values, and this trend became more pronounced with increasing PCS content, indicating that PCS promoted the early thermal decomposition of the WPU matrix. More importantly, the maximum decomposition rate of the first stage decreased significantly with increasing PCS content. This suggests that the amount of volatile decomposition products released during the first degradation stage was substantially reduced, and a greater fraction of carbon was retained in the condensed phase to form char.

The enhanced char formation during the early stage exerted a pronounced effect on the high-temperature degradation behavior. The second maximum decomposition rate (*T_d_*_2*max*_) shifted to higher values with increasing PCS content, and the corresponding decomposition rate was further suppressed. These observations indicate that the char formed during the initial decomposition stage provided improved resistance to thermal degradation at elevated temperatures. Consistently, the residual char yield (*R*_700_) increased dramatically from 0.7 wt% for neat WPU to 21.7 wt%, 26.7 wt%, and 32.7 wt% for WPU/PCS-3, WPU/PCS-4, and WPU/PCS-5, respectively. The simultaneous increase in *T_d_*_2*max*_, the slower decomposition rate in the second stage, and the much higher amount of char residue collectively demonstrate that PCS effectively promotes char formation in the condensed phase. During heating, the phosphorus-containing species generated from the phosphite groups of PCS catalyze dehydration and carbonization reactions, while the nitrogen-rich chitosan backbone participates in cross-linking, which further convert more carbon into a thermally stable solid residue rather than combustible gaseous volatiles. This pronounced char-forming effect is critical for improving the flame retardancy of the composite films.

### 3.4. Flame Retardancy of WPU/PCS-x Films

**Figure 5:**
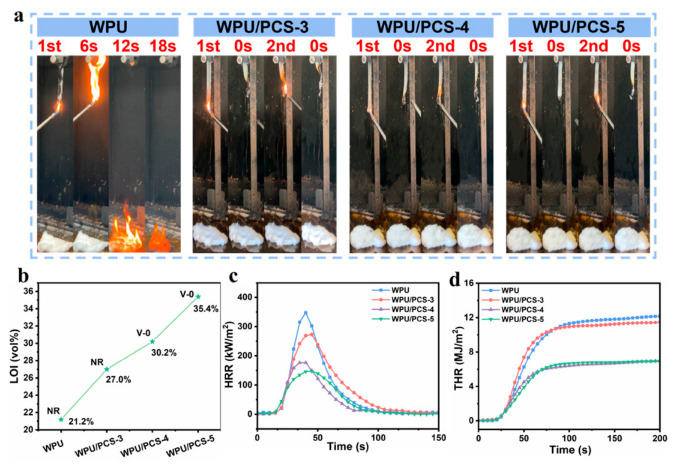
(**a**) Screenshot of UL-94 test video of WPU/PCS-x films, (**b**) UL-94 classifications and LOI values of WPU/PCS-x films, (**c**) HRR curves and (**d**) THR curves obtained from cone calorimeter measurements.

The flame-retardant performance of the WPU/PCS-x composite films was evaluated by UL-94 vertical burning, LOI, and cone calorimetry [33]. As shown in Figure 5a and Table 3, neat WPU burned rapidly during the UL-94 test, accompanied by prolonged after flame and severe flaming drips that ignited the cotton indicator placed beneath the specimen, revealing the poor self-extinguishing ability and high risk of secondary ignition. After the incorporation of PCS, the burning behavior of the composite films was markedly improved. The flame intensity was visibly reduced, and melt dripping was effectively suppressed. Although WPU/PCS-3 still failed to achieve a UL-94 rating, its dripping no longer ignited the cotton, indicating that PCS first suppressed the dripping hazard. With increasing PCS content, both WPU/PCS-4 and WPU/PCS-5 achieved a UL-94 V-0 rating with rapid self-extinguishing after each flame application and no flaming drips during the test. Moreover, the LOI value increased substantially from 21.2% for neat WPU to 35.4% for WPU/PCS-5, further confirming the substantially reduced flammability of the composite films.

Cone calorimetry was employed to further quantify the suppression effect of PCS on the heat release behavior of WPU. Neat WPU exhibited a high peak heat release rate (pHRR) of 347.8 kW/m^2^ and a total heat release (THR) of 13.4 MJ/m^2^, indicating intense combustion. After PCS incorporation, both pHRR and THR decreased progressively, with WPU/PCS-5 showing the most significant reductions. Specifically, the pHRR and THR of WPU/PCS-5 decreased to 92.6 kW/m^2^ and 7.8 MJ/m^2^, corresponding to reductions of 73.4% and 41.8%, respectively, compared with neat WPU. Meanwhile, the average effective heat of combustion (Av-EHC) decreased from 34.5 MJ/kg for WPU to 14.5 MJ/kg for WPU/PCS-5. This marked decrease suggests that PCS not only suppressed the heat release intensity but also lowered the combustion efficiency of the volatile degradation products.

The improved flame retardancy is consistent with the char-promoting effect of PCS revealed by TGA. The phosphorus-containing groups in PCS facilitate dehydration and carbonization during thermal exposure, while the chitosan backbone participates in cross-linking reactions that stabilize the resultant char. This protective char layer acts as a physical barrier, hindering heat and oxygen transfer, reducing melt flow, and limiting the release of combustible volatiles. Therefore, the pronounced suppression of both heat release and melt dripping, combined with the high LOI and V-0 rating, demonstrates that PCS effectively enhances the fire safety of composite films.

**Figure 6:**
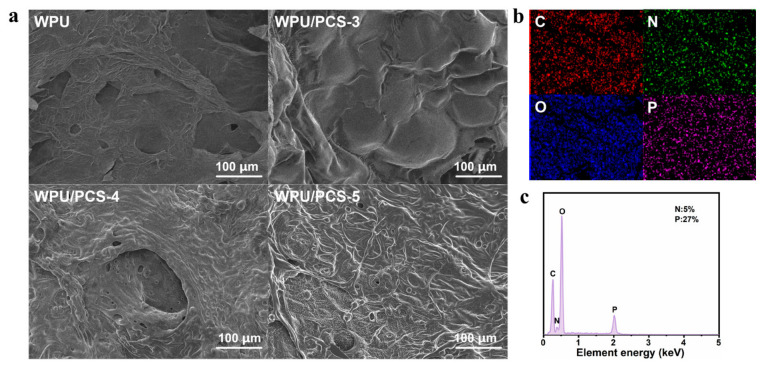
(**a**) SEM images of the char residues of WPU/PCS-x composite films, (**b**) elemental mapping images of WPU/PCS-5 and (**c**) EDS spectrum.

The morphology of the combustion residues was examined by SEM (Figure 6a). For neat WPU, the char residue was extremely thin, discontinuous, and exhibited pronounced cracks and voids, consistent with its low char yield. After incorporating PCS, the residues evolved progressively. For WPU/PCS-3, the char amount increased visibly; however, cracks and partial exfoliation were still observable, and the surface remained relatively flat but not fully continuous. For WPU/PCS-4, the char became more continuous with fewer cracks, and micron-sized cellular structures started to emerge. For WPU/PCS-5, the residue was complete, dense, and continuous, featuring uniformly fine cells without any through-thickness cracks. The char layer also adhered well to the underlying substrate. The corresponding EDS mapping (Figure 6b) and elemental analysis (Figure 6c) of the WPU/PCS-5 char surface revealed a phosphorus content of 27 wt% and a nitrogen content of 5 wt%. The high phosphorus retention confirms the strong condensed-phase char-forming ability of PCS, while the low nitrogen residue indicates that most nitrogen-containing species were released as non-flammable gases during combustion, contributing to gas-phase flame inhibition. These observations collectively demonstrate the P-N synergistic flame-retardant mechanism operating in both the condensed and gas phases.

### 3.5. Antibacterial Properties

**Figure 7:**
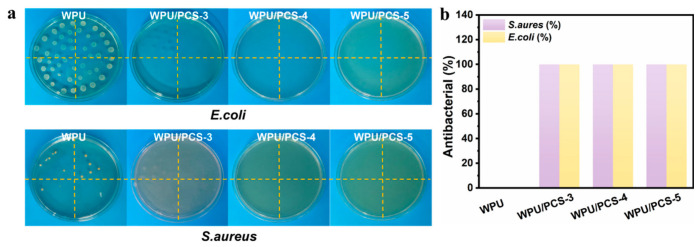
(**a**) Digital images of *E. coli* and *S. aureus* on WPU and WPU/PCS-x films, (**b**) antibacterial rates of the corresponding samples.

The antibacterial activity of the WPU/PCS-x composite films against *E. coli* and *S. aureus* was evaluated using the plate-counting method. As shown in Figure 7a, bacterial colonies were observed on the plates treated with neat WPU, indicating that the WPU matrix alone possesses negligible antibacterial activity. In contrast, after treatment with WPU/PCS-x composite films, almost no visible colonies appeared on the agar plates for either bacterial strain. Only faint residual marks caused by the evaporation of the bacterial suspension droplets could be observed on the agar surface, demonstrating that bacterial growth was effectively inhibited. The quantitative results in Figure 7b further confirm this observation. The antibacterial efficiencies of WPU/PCS-3, WPU/PCS-4, and WPU/PCS-5 against both *E. coli* and *S. aureus* were close to 100%, showing excellent broad-spectrum antibacterial activity. Moreover, a comparative analysis with previously reported antibacterial WPU systems was further conducted to verify the superiority of the WPU-PCS composite films. For instance, AgNPs-chitosan modified WPU exhibited antibacterial efficiencies of only 81.72% against *E. coli* and 83.27% against *S. aureus* [34], which were largely limited by the poor dispersion of inorganic components. This excellent antibacterial effect can be mainly attributed to the chitosan-derived PCS component. The protonated amino groups (-NH_3_^+^) of PCS can interact with negatively charged bacterial cell membranes through electrostatic attraction, disturbing membrane integrity and thereby suppressing bacterial proliferation [35,36]. Meanwhile, the uniform nanoscale dispersion of PCS with in the WPU matrix may facilitate the exposure of antibacterial active sites at the film surface. These results indicate that the incorporation of PCS effectively endows WPU with robust antibacterial functionality, further supporting the potential of WPU/PCS-x composites as multifunctional coating materials.

### 3.6. Evaluation of WPU/PCS-x as Flame-Retardant Coatings for Cotton Fabrics

**Figure 8:**
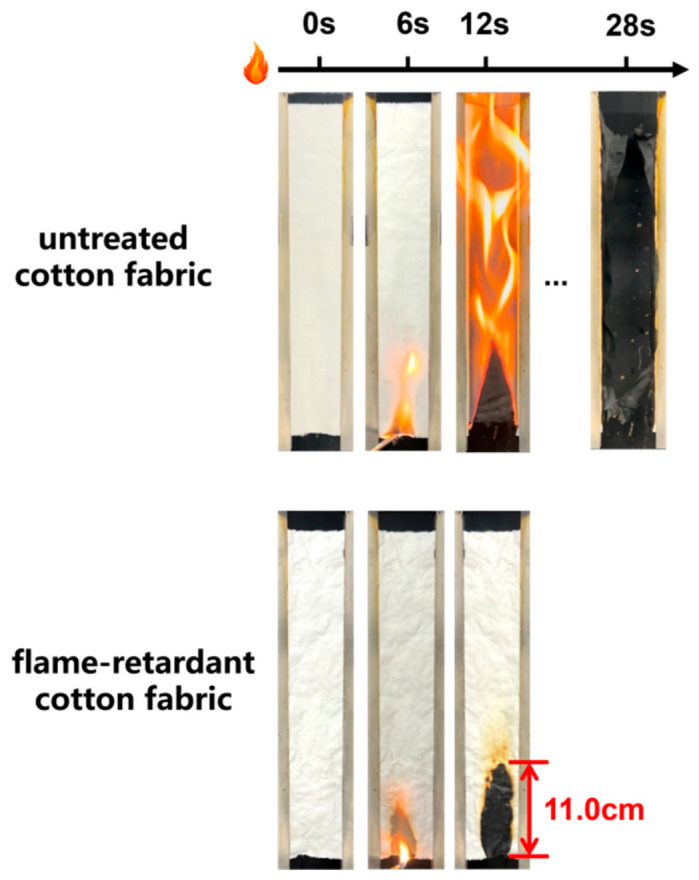
Digital photographs of the vertical burning test.

To assess the practical applicability of WPU/PCS-x system as a flame-retardant finishing agent for cotton fabrics, the WPU/PCS-5 emulsion was applied to cotton fabric, with neat WPU-coated fabric serving as the control. Vertical burning and LOI tests were performed, and the results are presented in Figure 8 and Table 4.

The vertical burning and LOI results clearly demonstrate the significant improvement in the flame-retardant performance of the WPU/PCS-5 coated fabrics. The cotton fabric coated with neat WPU was readily ignited after flame application, and the flame propagated rapidly upward along the fabric. Intense burning over a large area was observed at 12 s, and the fabric was completely consumed within 28 s, with a damaged length of 30.0 cm. Its LOI value was only 18.5%, indicating typical flammable behavior and insufficient fire resistance for flame-retardant applications. The cotton fabric coated with WPU/PCS-5, on the other hand, exhibited markedly improved fire behavior. After ignition, combustion was confined to the lower region of the specimen with no upward flame spread. Once the ignition source was removed, the flame self-extinguished immediately. The after-flame and after-glow times were both 0 s, and the final damaged length was reduced to 11.0 cm. Meanwhile, the LOI value increased significantly to 27.2%, representing a 47.0% improvement. The excellent coating performance of WPU/PCS-5 is directly linked to the char-forming ability of PCS and the structural integrity of the composite film. These results confirm that the WPU/PCS-x composite coating can effectively endow cotton fabrics with excellent self-extinguishing ability and resistance to flame propagation.

### 3.7. Comparison with Literature

To better evaluate the performance of our WPU/PCS-5 system, we compared it with representative flame-retardant and antibacterial waterborne polyurethane (WPU) composites reported in the recent literature. As summarized in Table 5, our system exhibits a LOI of 35.4% and a UL-94 V-0 rating, which are superior to most reported values. In addition, it shows no melt-dripping and achieves >99% antibacterial efficiency against *E. coli* and *S. aureus*. More importantly, the PCS is derived entirely from renewable chitin/chitosan, whereas most literature systems rely on petroleum-based or only partially bio-based components. This comparison clearly demonstrates that our bio-based modification strategy not only provides excellent fire safety and antibacterial functionality but also aligns with the principles of green and sustainable material design.

## 4. Conclusions

This work demonstrates that phosphorous acid-protonated chitosan (PCS) can serve as an effective emulsion-compatible, bio-based multifunctional modifier for cationic waterborne polyurethane. Despite broadening the particle-size distribution and inducing particle reorganization, the incorporation of PCS preserved the colloidal stability of the WPU emulsion, with zeta potential values remaining above 30 mV and no precipitation after centrifugation. PCS-rich nanodomains were uniformly dispersed within the WPU matrix, and the composite films retained high optical transparency. Rheological analyses revealed that the hydroxyl, protonated amino, and phosphite groups of PCS established a dynamic physical network with urethane segments through hydrogen-bonding and electrostatic interactions, which enhanced the viscoelastic response and structural integrity of the composite. This emulsion-compatible architecture translated into significantly improved fire safety. WPU/PCS-5 achieved a limiting oxygen index of 35.4% and a UL-94 V-0 rating, with peak heat release rate and total heat release reduced by 73.4% and 41.8%, respectively. The char residue at 700 °C increased from 0.7 wt% for neat WPU to 32.7 wt%, owing to the char-promoting effect of the phosphorus-containing chitosan structure. In addition, the composite films exhibited nearly complete antibacterial activity against *E. coli* and *S. aureus*, attributed to the protonated amino groups of the chitosan-derived PCS. When applied as a textile coating, WPU/PCS-5 enabled immediate self-extinguishing of cotton fabric, increased the LOI from 18.5% to 27.2%, and reduced the damaged length from 30.0 to 11.0 cm. This study provides a practical emulsion-compatible strategy for developing bio-based multifunctional WPU materials that integrate flame retardancy, anti-dripping behavior, antibacterial activity, and textile-coating applicability.

## Figures and Tables

**Table 1:** Centrifugation test and DLS test results for the WPU/PCS-x composite emulsion.

Sample	Average Size (nm)	PDI	Zeta Potential (mV)	Centrifugal Stability
WPU	39.38	0.310	34.2	No precipitate
WPU/PCS-3	99.86	0.534	37.7	No precipitate
WPU/PCS-4	95.81	0.498	39.1	No precipitate
WPU/PCS-5	94.63	0.565	35.8	No precipitate

**Table 2:** TGA test data of the WPU/PCS-x films.

Samples	*T*_5%_ (°C)	*T_d_*_1*max*_ (°C)	*T_d_*_2*max*_ (°C)	*R*_700_ (wt%)
WPU	231.3	276.6	359.3	0.7
WPU/PCS-3	163.0	226.8	362.2	21.7
WPU/PCS-4	156.2	217.5	362.8	26.7
WPU/PCS-5	149.3	211.7	363.6	32.7

**Table 3:** Cone calorimetry data and UL-94 ratings of WPU/PCS-x films.

Sample	PHRR (kW/m^2^)	THR (MJ/m^2^)	Av-EHC (MJ/kg)	UL-94 Rating	Dripping	Residues (wt%)
WPU	347.8	13.4	34.5	NR	Yes	3.8
WPU/PCS-3	277.1	13.0	28.6	NR	No	19.8
WPU/PCS-4	181.9	9.5	27.4	V-0	No	18.1
WPU/PCS-5	92.6	7.8	14.5	V-0	No	19.4

**Table 4:** Vertical combustion data and LOI values for fabric.

Samples	LOI (%)	After-Flame Time (s)	After-Glow Time (s)	Damaged Length (cm)
Untreated cotton fabric	18.5	28.0	0.0	30.0
Flame-retardant cotton fabric	27.2	0.0	0.0	11.0

**Table 5:** Performance comparison of WPU/PCS-5 with reported WPU composites.

Reference	Bio-Based Modification	Flame Retardancy		Dripping	Antibacterial Activity
		LOI	UL-94		
This work	Yes	35.4%	V-0	No	99.9% against *E. coli* and *S. aureus*
[37]	No	27.5% (coated fabric)	/	No	/
[38]	Yes	27.5%	V-0	No	99.9% against *E. coli* and *S. aureus*
[39]	No	22.9%	V-2	/	/
[6]	No	32.6%	V-0	No	/
[33]	No	/	/	/	81–84% against *E. coli* and *S. aureus*

## Data Availability

The data that support the findings of this work are available from the corresponding author upon reasonable request.

## Supplementary Materials

The following supporting information can be downloaded at: https://www.mdpi.com/article/10.3390/biomimetics11060384/s1, supplementary information regarding the formation mechanism of WPU/PCS composite system, viscosity of emulsion as a function of shear rate. (Figures S1 and S2).
