# Supplementary material for: A Multifunctional Cationic Waterborne Polyurethane System with High Fire-Safety and Antibacterial Performance Enabled by Phosphorous Acid-Protonated Chitosan
Source: Biomimetics (Basel). 2026 Jun 1;11(6):384. doi: 10.3390/biomimetics11060384 (PMC13297329; doi:10.3390/biomimetics11060384)
Supplement: Supplementary file 1 [file biomimetics-11-00384-s001.zip › biomimetics-4331869-supplementary.pdf]

Supporting information

A Multifunctional Cationic Waterborne Polyurethane System with High Fire-Safety and Antibacterial Performance Enabled by Phosphorous Acid-Protonated Chitosan

Xin-Yu Tian, Zhen-Guo Zhao, Peng Chen and Yan-Peng Ni *

Institute of Functional Textiles and Advanced Materials, College of Textiles & Clothing, Qingdao Key Laboratory of Flame-Retardant Textile materials, Shandong Key Laboratory of Polymeric Materials Recycling and Upcycling, National Engineering Research Center for Advanced Fire-Safety Materials D & A (Shandong), Qingdao 266071, China.

Correspondence: polyester-niyanpeng@qdu.edu.cn

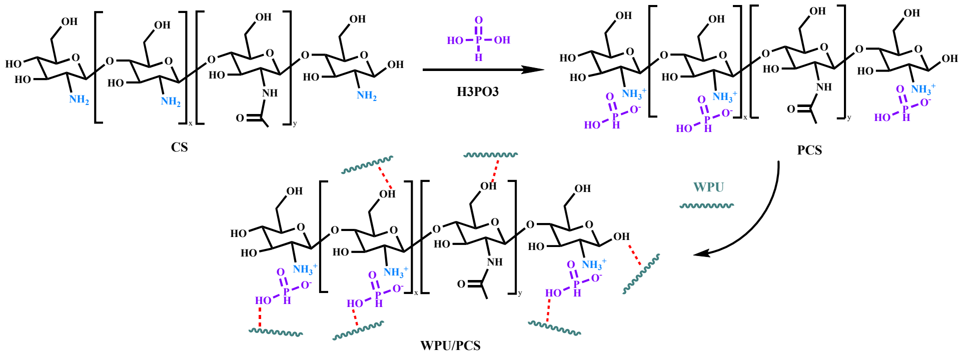

Figure S1. Formation mechanism of WPU/PCS composite system.

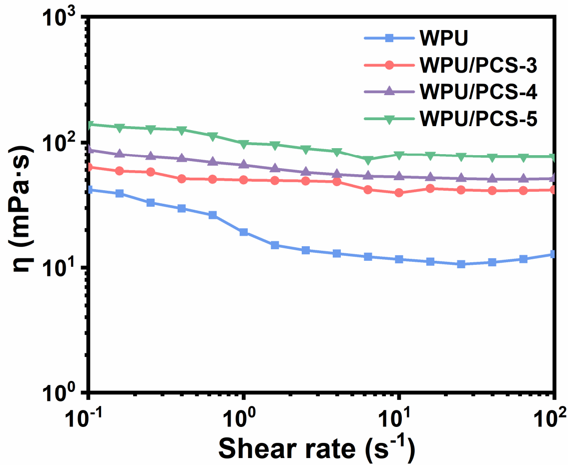

Figure S2. Viscosity of emulsion as a function of shear rate.
